# Numerical Simulation of Thermal Conductivity and Thermal Stress in Lightweight Refractory Concrete with Cenospheres

**DOI:** 10.3390/ma16010190

**Published:** 2022-12-25

**Authors:** Darius Mačiūnas, Szymon Nosewicz, Rimantas Kačianauskas, Renata Boris, Rimvydas Stonys

**Affiliations:** 1 Department of Applied Mechanics, Vilnius Gediminas Technical University, 10223 Vilnius, Lithuania; 2 Institute of Fundamental Technological Research Polish Academy of Sciences, 02-106 Warsaw, Poland; 3 Institute of Building Materials, Vilnius Gediminas Technical University, 10223 Vilnius, Lithuania

**Keywords:** thermal conductivity, heat transfer, finite element method, thermal stress, microstructure, calcium aluminate cement, cenosphere, refractory concrete

## Abstract

The main objective of this paper was to investigate the heat transfer of modified lightweight refractory concrete at the microscopic scale. In this work, such material was treated as a porous composite based on the compound of calcium aluminate cement and aluminosilicate cenospheres. The presence of air inclusions within the cenospheres was an essential factor in the reduction in thermal performance. Due to the intricacy of the subject investigated, our research employed numerical, theoretical, and experimental approaches. Scanning electron microscopy (SEM) imaging was performed to study the composite microstructure with a special focus on geometry, dimensions, and the distribution of cenospheres. Based on the experimental analysis, simplified geometrical models were generated to reproduce the main features of the composite matrix and cenospheres. A finite element framework was used to determine the effective thermal conductivity of such domains as well as the thermal stresses generated in the sample during the heat flow. A considerable difference in thermal properties was revealed by comparing the simulation results of the pure composite matrix and the samples, indicating a varying arrangement of cenosphere particles. The numerical results were complemented by a theoretical study that applied analytical models derived from the two-phase mixture theory—parallel and Landauer. A satisfactory agreement between numerical and theoretical results was achieved; however, the extension of both presented approaches is required.

## 1. Introduction

To meet the demand for energy savings in industrial furnaces, high-quality insulation materials characterized by high thermal resistance or low thermal conductivity must be applied. The issue of low-cost and eco-friendly thermal insulation materials has increased interest in exploring innovative solutions in such areas. 

Lightweight refractory composites with cenospheres generally meet the requirements of effective thermal insulation materials: high-temperature resistance, mechanical strength, low density, low shrinkage, eco-friendliness, and cost-effectiveness, all of which are mentioned in [1,2]. Cenospheres (CS) can be effectively incorporated into cementitious materials to produce lightweight refractory composites—such as ceramic matrix composites [3] and lightweight refractory concrete (LRC) [4,5]—and enhance thermal properties. A cenosphere is formed as a byproduct of coal combustion in thermal power plants [6]. It can be characterized by a hollow spherical particle with a stiff porous wall [7]. On average, the diameter of cenospheres (CSs) ranges between 30 and 350 μm, while the thickness of the porous wall increases linearly with the increase in the diameter of a CS particle and generally ranges between 2.5% and 10.5% of the diameter [8]. The strong wall of the CS minimizes the losses in strength and elastic modulus caused by the increase in the porosity of the lightweight refractory concrete samples. A CS provides the following exceptional properties to an LRC at high temperatures (up to 1400 ℃) [9]: high resistance to thermal shock [10], high porosity [11], ultra-low density [6], lightweight and excellent mechanical strength [12], low thermal expansion [1], resistance to acids and bases [13], good water absorption [14], resistance to oxidation, corrosion [15], and protection against electromagnetic interferences [1].

Despite its several advantageous properties, LRC suffers from thermal/residual cracking generally caused by external structural properties (thermal stresses, heating rate, constraints, and applied loads) and internal material properties (porosity, tensile strength, moisture content, and fiber content) [16,17]. For ultra-lightweight cement composites (ULCCs), the degree of thermal damage and crack formation increases with the rise in the exposure temperature [18].

Considering both the advantages and drawbacks of LRC, thermal conductivity seems to be a key factor and has the greatest impact on LRC materials’ performance due to their potential industrial applications. A comprehensive experimental study was carried out to estimate the effects of a CS on both the mechanical and thermal properties of ULCCs [19]. CSs were studied in connection with various sizes, proportions, and wall thicknesses. It was revealed that the thermal conductivity of ULCC mainly depended on the volume fraction of CS, while the mechanical properties of ULCC were governed by the size of the CS and the thickness of the wall. It was concluded that high thermal insulation and high-strength ULCCs could be manufactured by incorporating smaller-size CSs with strong shells. In addition, an analytical approach was suggested to estimate the thermo-mechanical properties of ULCCs. Low-thermal-conductivity and lightweight, hydrophobic, thermal-shock-resistant cement composites were developed by combining calcium aluminate phosphate cement as the binder and the fly ash cenosphere and hydrophobic silica aerogel as insulating particles [20]. As a result, the optimized composite samples, after thermal shock tests, obtained thermal conductivity values of 0.35 and 0.28 W/mK at 100 and 250 °C, respectively.

The thermal and mechanical characteristics of ULCCs and the inclusion of CSs have been experimentally investigated [21]. Microstructural analysis has shown the interactions between the cement matrix and CSs. An analytical method was introduced to assess the effective volumetric heat capacity of ULCCs with CSs, and the experimental and analytical results were compared. As a general conclusion, an analysis of the literature showed that the incorporation of cenospheres in concrete leads to a significant lowering of the values of the thermal conductivity coefficient [22,23]. In [24] the creep behavior of both slender aged and short reinforced concrete columns with a sustained load capacity of more than 90% of the column capacity was investigated. The confinement effects of transverse reinforcement reduced both the creep deformation and the risk of column failure under continuous loads. This conclusion is very useful for testing and analyzing experimental results. It provides a proper interpretation of the phenomena indicated in the simulations of our paper.

Apart from the presented experimental investigations, the thermal conductivity of porous composites, such as LRC, can be evaluated using several modeling methods depending on the approach used to incorporate composite microstructures. In terms of thermal-conductivity phenomena, every examined characteristic of a microstructure may be generalized by the effective thermal conductivity that represents the macro reaction of the entire porous composite. Numerous models have been derived to predict the effective thermal conductivity of two-component composites, as reviewed in [25]. Generally, a two-phase mixture system is the simplest in composite materials [26]. In comparison, few models exist for three-component composites [23,27,28].

Specifically, the thermal conductivity of the ULCC was evaluated using the Hashin–Shtrikman lower bound [23]. An investigation showed that the thermal conductivity of a ULCC that incorporated a 0.5 volume fraction of CS was reduced by 80% compared to the conventional cementitious composite. In [29], the thermal conductivity of the ULCC was reduced by 80% by incorporating 42 wt% CS.

The boundary condition plays an important role in predicting the effective thermal conductivity of composites. In [30], the periodic boundary condition and the volume-representative element-based FE homogenization method were adopted to evaluate the effective thermal conductivities of the composites reinforced by the spherical, ellipsoidal, and cylindrical inclusions. A particular set of porous composites were prototypes manufactured through heating techniques [31,32]. An extensive review [33] included semi-empirical models to measure the thermal conductivity of particle-filled composites. Most of the aforementioned works included semi-empirical methods to predict effective thermal conductivity. The spatial-partitioning approach based on Voronoi tessellation was used to determine three different gradient Voronoi random structures to improve the adaptability of a thermal protection system [34]. The effective thermal conductivity of the porous spatial Voronoi gradient structure was affected by the regularity and orientation of the porous structure.

Basically, the thermal properties of the LRC are predicted by its microstructure. However, a precise experimental evaluation of the microstructure is too costly. On the other hand, theoretical predictions of the thermal conductivity of the porous composite have a considerable degree of indeterminacy due to an excessively simplified physical microstructure of the porous composite. Since precise knowledge of the heat-transfer mechanism seems to be a key factor in the explicit evaluation of thermal conductivity in LRC, a numerical simulation approach was suggested to develop a deeper and more precise understanding of the realistic representations of the heat-transfer processes. 

Recently, numerical simulations of the heat transfer of a porous composite with regard to microstructure have become a suitable alternative for evaluating the thermomechanical behavior [35,36]. A finite element simulation of the volume-representative element with periodic boundary conditions was performed to characterize the microstructure of a polymer matrix reinforced with cenospheres [37]. The study involved the application of the random sequential adsorption theory to define the distribution of the cenospheres. The micromechanical behavior was investigated; however, an analysis of thermal effects was not included in the paper. To explore the mechanical response in ultra-lightweight cement composites (ULCC) when cenospheres are introduced into the matrix, a numerical simulation based on the finite element method (FEM) was performed [38]. Stress distributions in ULCC with and without cenospheres revealed that stress concentrations occurred in the shell layer of the cenospheres. However, thermal behavior was not included in the numerical simulation.

Considering the advantages and drawbacks of models found in the literature, this work introduced a FEM-based numerical model to investigate the influence of cenospheres on the thermal properties of LRC. In the current literature, we noticed that many aspects of numerical simulation of the thermal conductivity of LRC have not yet been explored or are rare.

The purpose of this work was to perform numerical simulations and investigate the influence of cenospheres on the thermal properties of LRC at elevated temperatures. We aimed to numerically evaluate the thermal conductivity of LRC, compare numerical results with the analytical solution, and predict the location of thermal stress in the composite. The present paper aimed to utilize the numerical modeling of thermal conductivity based on the framework of finite elements employing COMSOL multiphysics. The numerical results of the effective thermal conductivity were accompanied by analytical results of the two simple theoretical relationships based on the two-phase combination theory. This work is the first step in the numerical comparative investigation of the thermo-mechanical characteristics of LRC incorporated with CSs.

## 2. Materials and Methods

In the present work, a particulate alumina-based lightweight refractory composite (ALRC) was selected as the representative material for thermal conductivity analysis and thermal stress prediction. Such ALRCs may withstand temperatures up to 1800 °C [39] due to the particular properties of the chosen constituent calcium aluminate cement (CAC). The ALRC was manufactured by hardening under normal conditions and the thermal treatment of the samples [31,32]. The morphology of the materials involved and the manufacturing conditions (heating temperature, time, atmosphere, assisted pressure, heating, and cooling rate) should be considered the most important factors affecting the density and porosity of the ALRC manufactured.

**Figure 1:**
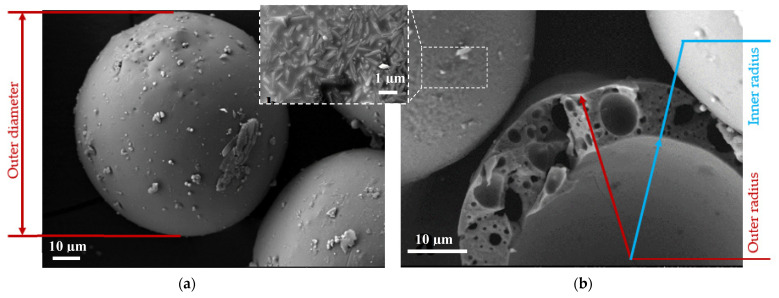
Microstructure and phase analysis of the aluminosilicate cenospheres: (**a**) Outer view (including crystalline structures at 1 μm scale); (**b**) Inside view (inner radius of internal pore and closed pores inside the wall of the cenosphere).

In the present article, the constituent materials for the composite mixes included CAC (Górkal70 produced by Górka Cement Company, Poland: Al_2_O_3_ > 70%, with a specific surface area of 450 m^2^/kg) and aluminosilicate CS (Al_2_O_3_ comprised 34–38% and SiO_2_ comprised 50–60% of the composition, with an outer diameter ranging from 50 to 150 μm) (Figure 1a). The microstructure of aluminosilicate CS is usually defined as a hollow microsphere (Figure 1) and a mixture of amorphous phases and different crystalline structures, such as mullite, magnetite, periclase, quartz, and lime [40].

**Figure 2:**
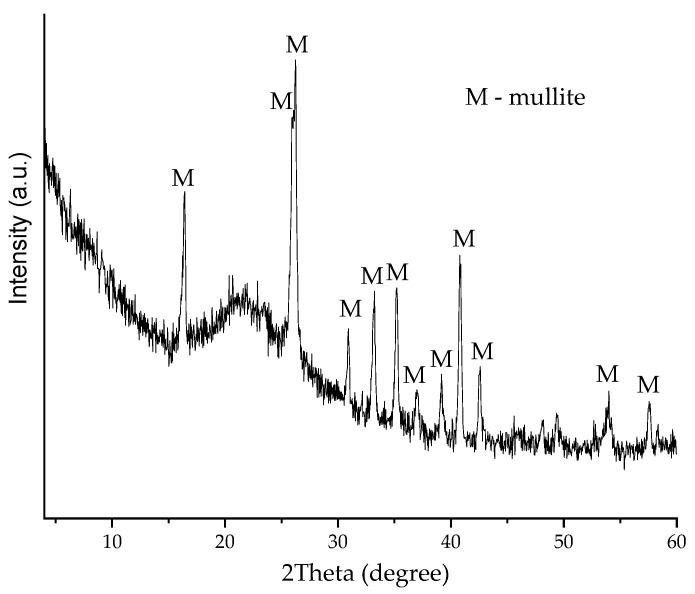
XRD difractogram of the aluminosilicate cenospheres (M-mullite).

X-ray diffraction (XRD) analysis showed (Figure 2) that the wall of the CS mainly consists of mullite (3Al_2_O_3_⋅2SiO_2_). XRD analysis was performed using a DRON-7 diffractometer (St. Petersburg, Russia) with Cu-Kα (λ = 0.1541837 nm) radiation. The following test parameters were applied: 30 kV voltage, 12 mA current, and a 2θ diffraction angle range from 4° to 60° with an increase of 0.02° measured each 2 s. The XRD diffraction fractions were identified by comparing the diffraction spectrograms with standard diffraction patterns provided by the International Centre for Diffraction Data (ICDD). The XRD pattern indicated a crystallization process with an amorphous phase and a crystalline phase [41].

The water-to-cement ratio was chosen in accordance with common technical requirements to ensure proper hydration of the cement mass [26,39,42]. In the ALRC formulation, the water dosage was adjusted to achieve proper fluidity and workability for mold casting. The proportion of materials in the mix is provided in Table 1.

The mixing procedure for the preparation of the cement paste was as follows: first, dry components (calcium aluminate cement and aluminosilicate cenospheres) were mixed in the Hobart-type mixer for 5 min; then, water was poured into the mix; finally, all constituent materials were mixed in the Hobart-type mixer for the next 5 min. All samples were formed, cured, and heated in accordance with the LST EN ISO 1927-5:2013 standard [43].

**Figure 3:**
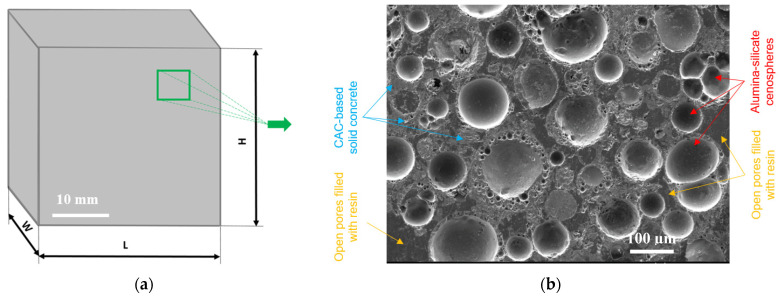
ALRC sample: (**a**) Cube-shaped L × W × H = 40 × 40 × 40 mm; (**b**) Microstructure SEM image.

The final size of the cube-shaped ALRC samples (L × W × H) was 40×40×40 mm, respectively (Figure 3a).

Microstructure analysis was performed using the JSM-7600F scanning electron microscope (SEM) (JEOL, Tokyo, Japan). The analysis was carried out at an acceleration voltage of 10 kV; the mode of secondary electrons was used in image formation. Before the investigation, the surface of small pieces of specimens to be investigated was covered with a layer of electricity-conducting material using the device QUORUM Q150R ES (Quorum Technologies Ltd., Reutlingen, Germany). Small pieces of paste were taken.

The SEM image showed clearer boundaries between the aluminosilicate CS, the CAC-based solid concrete, and the two-component resin used to fill the open pores (in solid concrete) to harden the samples before impregnation, cutting, grinding, and polishing (Figure 3b). The thickness of the porous wall of CS mainly varied between ~1 and 15 μm. The resin, a non-porous part of a uniform-density glassy texture, filled pores and gaps in the ALRC. CAC-based concrete is a porous grayish-colored material that surrounded the CSs and incorporated them into the ALRC. A SEM image (Figure 3b) revealed the chaotic distribution of the CS in the ALRC sample with an average distance between the centers of adjacent CSs in the range of 50–100 μm.

The porosity of the ALRC comprises two fractions [44]:

- Effective porosity (also known as open porosity) is described by the total volume of open pores in CAC-based solid concrete filled with resin (Figure 3b).
- Ineffective porosity (also known as closed porosity) is described by (1) the internal pore volume of the cenosphere (predetermined by the inner radius) (Figure 1b); (2) the total volume of closed pores inside the wall of the cenosphere (Figure 1b).

To understand the heat-transfer process in the ALRC, a series of numerical experiments was performed. Since cenospheres were used to model the porosity of ALRC, only the internal pore volume of the cenosphere was included in the numerical simulation (Figure 1b).

### 2.1. Theoretical Foundations of Heat Transfer: Thermal Conductivity and Thermal Stress

The transfer of heat defines the exchange of thermal energy and the distribution of temperatures, including the alterations caused by temperature gradients. Thermal energy flow is divided into three processes: radiation, conduction, and convection. These processes can occur simultaneously, regardless of their different properties. Conduction involves the transfer of heat via the excitation of atoms. Convection involves the transfer of heat through the movement of molecules caused by differences in temperature. However, radiation involves the transmission of heat via electromagnetic energy. Radiative transfer of heat and convection occur through pores in a porous medium but are limited in intensity due to the restricted thermal flow potential. In solid materials, the most dominant heat-transfer process is thermal conduction.

The conduction of heat is composed of electron movement and lattice vibration [45]. In cement-based materials, heat transfer usually occurs electronically, while the lattice component is generally minor [44]. The transfer of heat through insulating materials occurs mainly due to lattice vibrations [46]. The ALRC investigated in this work might include one of the above-mentioned behaviors or their arrangement. The ratio between phonons or electronic mechanisms is determined by several characteristics, e.g., porosity and phase distributions, electronic and crystal structure, temperature, particle size, impurity levels, and chemical and phase composition [47,48].

Mathematical analysis of heat conduction shows that the heat flux is proportional to the negative temperature gradient and thermal conductivity, as defined by the Fourier law [49].

Effective thermal conductivity may define the average thermal properties of heterogeneous materials. The effective thermal conductivity (i.e., $\lambda_{eff}$) of porous materials can be determined by replacing the local thermal conductivity with the effective global coefficient $\lambda_{eff}$ defining the spatially averaged heat transfer. $\lambda_{eff}$ evaluates the disturbance of the heat transfer, associated not only with various thermal characteristics of the sample components but also with the occurrence of pores, inclusions, particle boundaries (PB), and other sources [50]. There is a strong correlation between the thermomechanical process and the temperature field in concrete-based materials [51]. Temperature gradients trigger thermal expansion gradients, causing tensile stresses to be perpendicular to the heated surface.

### 2.2. Analytical Methods for the Transfer of Heat and Effective Thermal Conductivity of Porous Materials

Analytical methods of estimating effective thermal conductivity in terms of material porosity consider the combination of two phases: solid and pores. These methods disregard radiation and denote only heat transfer within solid and gas phases. The choice of an appropriate method depends on the shape of the pores, the porosity range, the topology of the pores, and other microstructural characteristics [50]. Several analytical methods are described further for porous materials with pore volume fractions between 0.65 and 1.00.

The simplest expression that considers the volumetric law of mixtures averages the effective conductivity $\lambda_{eff}$ as follows [52]:

(1) $$\lambda_{eff}=\lambda_{s}\left(1-v_{p}\right)+v_{p}\lambda_{p},$$

where $\lambda_{s}$ is the thermal conductivity of the solid, $\lambda_{p}$ is the thermal conductivity of the pores, and $v_{p}$ is the pore volume fraction. Equation (1) sets an upper limit to the arrangement of the two phases.

For porous materials with pore volume fractions up to 0.65, the Landauer relationship (Equation (2)) has become the standard analytical method to estimate effective thermal conductivity. The method predicts heat transfer in solid or porous materials—for instance, in nanostructures [53]. Equation (2) was derived from the analysis of heat-conduction phenomena through porous materials with a random distribution of both phases, including the presence of open porosity connecting the numerous networks:

(2) $$\lambda_{eff}=0.25\left[\lambda_{p}\left(3v_{p}-1\right)+\lambda_{s}\left(2-3v_{p}\right)+{\left\{{\left[\lambda_{p}\left(3v_{p}-1\right)+\lambda_{s}\left(2-3v_{p}\right)\right]}^{2}+8\lambda_{s}\lambda_{p}\right\}}^{0.5}\right].$$

Analytical expressions (Equations (1) and (2)) were used to validate the results obtained by numerical simulations in COMSOL.

### 2.3. Finite Element Method Based on the Numerical Model in COMSOL

The numerical simulation of thermal conductivity and thermal stress of ALRC samples was implemented by finite element analysis (FEA) based on the numerical model in the computer aid engineering (CAE) software COMSOL Multiphysics (COMSOL Inc., Stockholm, Sweden). The main goal of FEA of heat conduction is to calculate the thermal conductivity of the ALRC sample, together with the prediction of thermal stress on the microscopic scale. The numerical procedure consisted of three main steps: (1) the creation of the finite element model on COMSOL, (2) the solution of the thermal problem on COMSOL, and (3) the calculation of the thermal conductivity on the resultant heat fluxes (temperatures), including the prediction of thermal stress.

**Figure 4:**
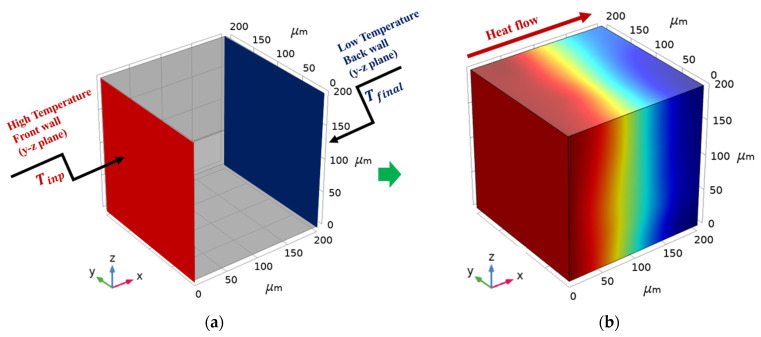
The 3D simulation model: (**a**) Boundary conditions; (**b**) Heat flow.

The first step consisted of creating the geometry of the ALRC model, including the assignment of material and boundary conditions. Then, COMSOL provided a finite element mesh in accordance with user settings. Hence, the spatial distribution of the elements represented a real structure of the sample. The boundary conditions were related to the temperature on the front and back walls of the sample, assumed to be 1250 ℃ and 1400 ℃, respectively. The remaining walls of the sample completely blocked out the heat flow that was normal to these surfaces by default, equivalent to its adiabatic behavior (Figure 4). Additionally, appropriate thermal properties related to all materials in the ALRC were assigned. A nonzero thermal conductivity value through the pores was set to avoid singularity.

The contribution of meshing, temperature change, time step, and specified size and location of the cenospheres (topology) to the thermal-conductivity value was considered in this paper.

To define the thermal conductivity of the composite along with the prediction of thermal stress, the time-dependent thermal-conduction problem was considered. The numerical setup was arranged analogously to the physical experiment in accordance with ISO 8894-1:2010. To study the thermal conductivity of the ALRC microstructure, a temperature difference was applied on the opposing sides of the 3D solution domain (unit cell/model), with the remaining sides subjected to (adiabatic) boundary conditions. A constant temperature ($T_{inp}$ and $T_{ref}$) was applied on the 3D solution domain (unit cell) to indicate the applied boundary conditions for calculating thermal conductivity (Figure 4). $T_{ref}$ considered the initial temperature of the 3D solution domain while $T_{inp}$, the input temperature, was applied on the front wall. Due to the temperature difference, a heat flux occurred from the high-temperature bound towards the low-temperature bound.

The geometry of the 3D solution domain was defined in Cartesian coordinates Oxyz by the specified length *L* and the squared cross-section with the edge length *h*. The thermal flow was restricted to a one-dimensional case. The thermal input was given by the temperature $T_{inp}$, which was specified on boundary *x* = 0 at time instant $t_{0}=0$. The walls parallel to the Ox axis were defined as perfect insulation. The thermal output was specified by the variation in the outlet temperature at the boundary *x* = *L* at time instant $t=t_{final}$. Temperature $T_{final}$, stationary temperature, was reached at time instant $t_{final}$. A fixed constraint was applied on the front wall’s y–z plane to simulate stress in the composite.

Thermal conductivity was estimated considering the temperature differences obtained by solving the uniaxial time-dependent thermal flow problem as follows [54]:

(3) $$\lambda =\frac{q}{4\pi }\cdot \frac{\ln\left(t_{2}/t_{1}\right)}{\Delta T_{2}-\Delta T_{1}},$$

where q is the heat flux per unit length along the longitudinal axis of the model, and $\Delta T_{1}$ and $\Delta T_{2}$ are the temperature changes defined at specified time instants $t_{1}$ and $t_{2}$, respectively.

## 3. Results

This paper presents the results of the numerical heat-transfer analysis of refractory concrete samples subjected to elevated temperatures (up to 1400 ℃) similar to the temperatures occurring in the furnace. An extensive numerical analysis of the heat transfer of the samples was carried out based on the methodology presented in Section 2. Three geometric models representing the ALRC samples with different microstructures were used in finite element simulations to evaluate thermal conductivity and its dependence on structural and thermal features.

In this section, a description of the experimental results is provided as follows:

- Homogeneous model;
- One-particle model of the composite;
- Model of two particles of the composite.

Then, the interpretation of the results, as well as the experimental conclusions, are drawn.

To illustrate our methodology, several testing samples were solved. The thermal input data were defined from the experiment in [54], where the input temperature $T_{inp}=1400\;℃$ (Figure 4a) was generated by heat flux. The reference temperature $T_{ref}$ was equal to 1250 ℃. Thermal insulation was applied to the lateral walls. The output temperature $T_{final}$ (Figure 4a) was defined as the average temperature on the back wall. Finally, the thermal conductivity was obtained by Equation (3) using $\Delta T_{1}$ and $\Delta T_{2}$ at time instants $t_{1}=0.0025\;s$ and $t_{2}=0.01\;s$, respectively.

### 3.1. Homogeneous Models

#### Calcium Aluminate Cement, Aluminosilicate Cenospheres, and Air

As indicated above, as the first step of the numerical simulation, the finite element model of homogeneous materials (MHM) on COMSOL was created as a cube-shaped sample (L × W × H) of 200×200×200 μm (Figure 4) for each of the materials (Table 2):

- Model of homogeneous material 1 (MHM1): the shell of the aluminosilicate cenosphere;
- Model of homogeneous material 2 (MHM2): calcium aluminate cement;
- Model of homogeneous material 3 (MHM3): the air inside the aluminosilicate cenosphere.

**Figure 5:**
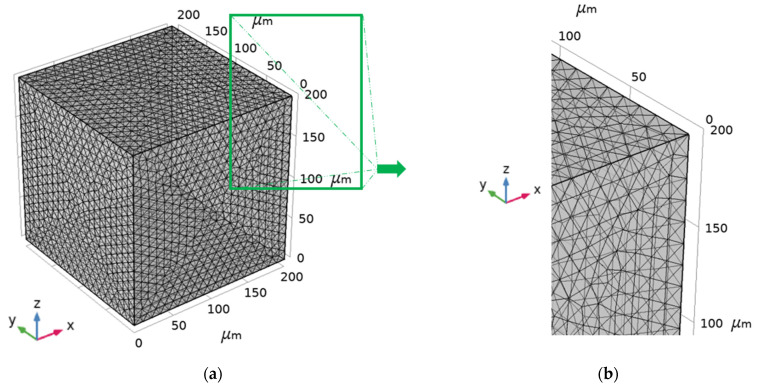
Mesh of simulated FEM model: (**a**) Whole; (**b**) Fragment.

The FEM model was generated using an unstructured mesh containing 100,524 tetrahedral elements (Figure 5) for each MHM. Based on multiple numerical tests, the mesh size was chosen to obtain accurate results.

The simulation value of thermal conductivity $\lambda_{si}$ (calculated using Equation (3)) of each MHM was compared with the corresponding input value of thermal conductivity $\lambda_{in}$ to observe the sufficiency of FEM modeling. Close agreement of the simulated thermal conductivity $\lambda_{si}$ of each MHM (MHM1, MHM2, and MHM3) with the corresponding input values $\lambda_{in}$ demonstrates the suitability of the modeling and sufficient accuracy (Table 3).

### 3.2. Models of Composites 1, 2, and 3

The properties of the constituent materials of ALRC (Composites 1, 2, and 3) are shown in Table 2.

**Figure 6:**
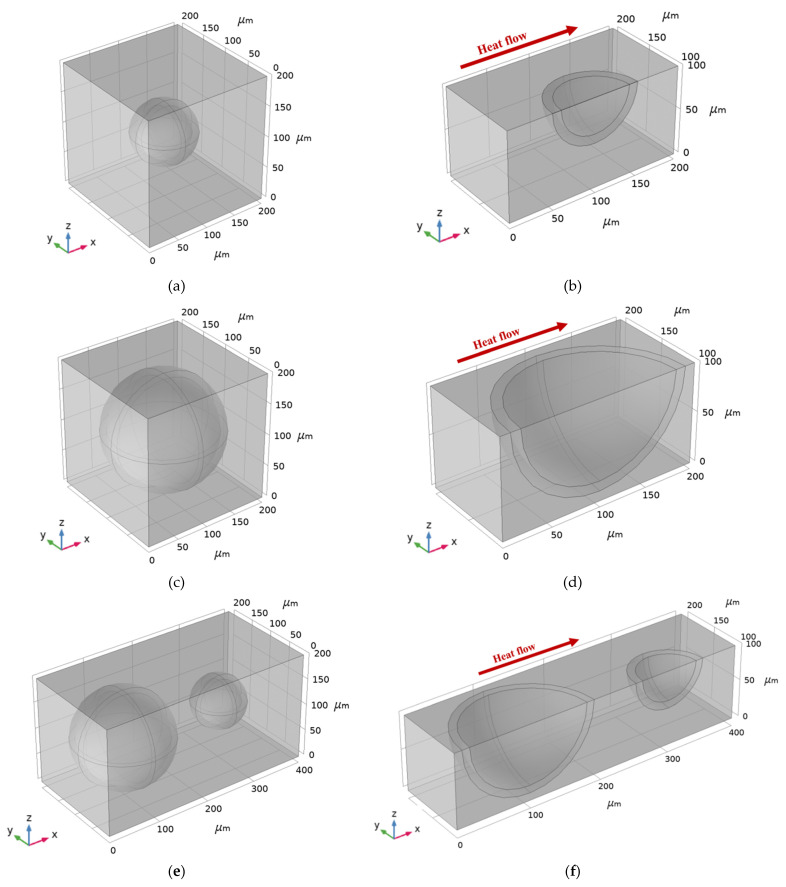
Model of composites: (**a**) Composite 1 with ∅80−∅100 μm cenosphere inside of the 200×200×200 μm cube; (**b**) One-quarter of the model of Composite 1; (**c**) Composite 2 with ∅160−∅180 μm cenosphere inside of the 200×200×200 μm cube; (**d**) One-quarter of the model of Composite 2; (**e**) Composite 3 with ∅80−∅100 μm and ∅160−∅180 μm cenospheres inside of the 400×200×200 μm cube; (**f**) One-quarter of the model of Composite 3.

Composite 1 contained a cenosphere with inner and outer diameters of ∅80−∅100 μm, respectively (Figure 6a), centered in the cube-shaped sample with dimensions (L × W × H) of 200×200×200 μm, respectively; Composite 2 contained a cenosphere with inner and outer diameters of ∅160−∅180 μm, respectively (Figure 6c), centered in a cube-shaped sample with dimensions (L × W × H) of 200×200×200 μm, respectively; and Composite 3 contained two cenospheres with inner and outer diameters of ∅160−∅180 μm and ∅80−∅100 μm, respectively (Figure 6e) in a cube-shaped sample with dimensions (L × W × H) of 400×200×200 μm, respectively. For modeling purposes, the air inside the cenosphere was considered, although flue gas was generally trapped inside the cenosphere [1]. Table 3 shows a correlation between the volume fraction (VF) of the constituent materials and thermal conductivity in each composite model.

While modeling a quarter of a 3D solution domain of each ALRC (Composite 1, Composite 2, and Composite 3), in addition, two symmetry planes (x-y and x-z) were considered as boundary conditions (Figure 6b,d,f). One-quarter of the model obtained accurate results while saving computational resources.

**Figure 7:**
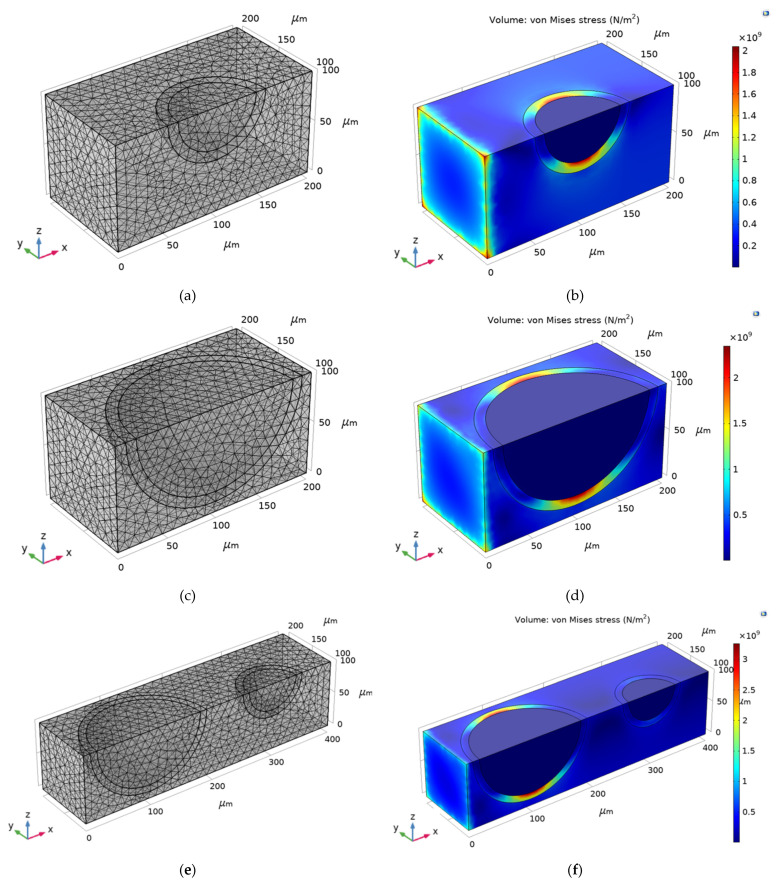
Model of composites: (**a**) Mesh of Composite 1; (**b**) von Mises stress of Composite 1; (**c**) Mesh of Composite 2; (**d**) von Mises stress of Composite 2; (**e**) Mesh of Composite 3; (**f**) von Mises stress of Composite 3.

The FEM model was generated by an unstructured mesh containing the following:

- 24,552 tetrahedral elements in Composite 1 (Figure 7a);
- 23,297 tetrahedral elements in Composite 2 (Figure 7c);
- 24,829 tetrahedral elements in Composite 3 (Figure 7e).

As the temperature increased, the von Mises stress also increased, thus damaging the corresponding composites (Figure 7b,d,f) due to the stress concentration at the layer of the shell of a cenosphere. One possible explanation for this concentration is that damage to the interface transition zone between the particle and the cement paste caused the micro-cracks in the composites to increase [56].

**Figure 8:**
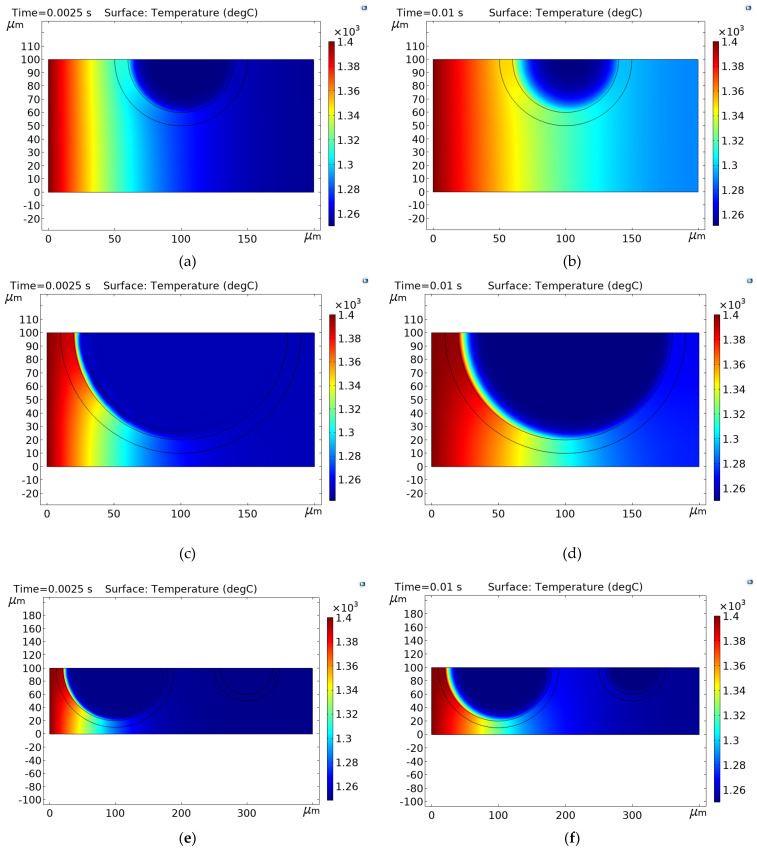
Graphical distribution of the temperature magnitudes in: (**a**) Composite 1 over x-z plane at time instant $t_{1}$; (**b**) Composite 1 over x-z plane at time instant $t_{2}$; (**c**) Composite 2 over x-z plane at time instant $t_{1}$; (**d**) Composite 2 over x-z plane at time instant $t_{2}$; (**e**) Composite 3 over x-z plane at time instant $t_{1}$; (**f**) Composite 3 over x-z plane at time instant $t_{2}$.

As reported in recent research, model samples containing cenospheres resulted in a minimal open porosity [57], and the open pores were not interconnected [38]. Therefore, the influence of cenospheres (i.e., closed porosity) on the thermal properties of the ALRC is crucial. With a representation of the temperature distribution on the surface of the x–z plane, it is possible to characterize the local thermal effects of the ALRC. Figure 8 represents the graphical distribution of the temperature (T) magnitudes of the samples at time instants $t_{1}=0.0025\;s$ and $t_{2}=0.01\;s$ for Composite 1 (Figure 8a,b), Composite 2 (Figure 8c,d), and Composite 3 (Figure 8e,f). At time instants $t_{1}=0.0025\;s$ and $t_{2}=0.01\;s$, the largest temperature-distribution rate was observed in Composite 1 (Figure 8a,b, respectively).

It is important to note that the temperature distribution rate was significantly lower in the cenosphere air pore compared to the rest of the sample in Composite 1 (Figure 8a,b), Composite 2 (Figure 8c,d), and Composite 3 (Figure 8e,f). The thermal conductivity of air was significantly lower than that of other materials of the ALRC (Table 3); therefore, the thermal resistance of the cenosphere air pores was significantly higher due to the effect of closed porosity. Figure 8 shows that the higher volume fraction of air $VF_{Ai}$ in the composite (Table 3) leads to a lower temperature-distribution rate in the sample along the *x*-axis. On the other hand, the location of CSs and the length of the composite samples also play a significant role in temperature distribution over the x–z plane (Figure 8). This observation explains the difference in the simulated thermal conductivity values $\lambda_{si}$ between Composite 1, Composite 2, and Composite 3 (Table 3). The comparison of Composite 1, Composite 2, and Composite 3 shows that the maximal volume fraction of air $VF_{Ai}$ is in Composite 2 (i.e., 0.2681), and the minimal $VF_{Ai}$ is in Composite 1 (i.e., 0.0335). Therefore, the maximal volume fraction of air in Composite 2 resulted in a minimal simulated thermal conductivity value ($\lambda_{si}=0.4215$) compared to Composite 1 and Composite 3 ($\lambda_{si}=0.6274$ and $\lambda_{si}=0.5218$, respectively).

A slight disagreement between the simulated values $\lambda_{si}$ and the effective values $\lambda_{eff}$ of thermal conductivity in composite-type samples (Composite 1, Composite 2, and Composite 3) can be explained by the fact that Equations (1) and (2) do not consider the actual position of cenospheres in the composite (Table 3). Since the numerical models evaluate thermal conductivity while considering the location of CS in each composite, thermal conductivity $\lambda_{si}$ is evaluated more accurately compared to analytical approach $\lambda_{eff}$.

The three composite-type samples (Composite 1, Composite 2, and Composite 3) demonstrated the application of the FEM particle model on composites. The numerical model introduced in this paper enabled us to investigate the influence of cenospheres on the thermal properties of the ALRC with greater precision.

## 4. Discussion

The main objective of this paper was to determine the influence of cenospheres on the thermal conductivity value of ALRC and numerically simulate the thermal conductivity $\lambda_{si}$ of the composite samples and compare the results with analytical solution $\lambda_{eff}$; in addition, we aimed to determine the location of thermal stress in the composite.

Heat conduction in ALRC is the combination of the thermal conduction of the solid phase of the matrix and the thermal conduction of the fluid or gas phase trapped in the pores. The incorporation of cenospheres into the ALRC led to an increase in the air voids in the ALRC. Therefore, the thermal conduction surface of the solid phase decreased, whereas that of the gas phase increased. 

Cenospheres have a significant effect on the thermal properties of the ALRC. The incorporation of cenospheres into the cementitious matrix resulted in a 20% to 25% decrease in the thermal conductivity of the LRC [21]. Table 3 shows that the decrease in the thermal conductivity $\lambda_{si}$ of the ALRC was 13.03% (Composite 3) and 29.75% (Composite 2) compared to thermal conductivity $\lambda_{si}=0.61$ of MHM2. Lower thermal conductivity values have been observed in ALRCs with a larger volume fraction (VF) of air: the thermal conductivity of Composite 3 ($VF_{Ai}=0.1508$) and Composite 2 ($VF_{Ai}=0.2681$) was 0.5218 and 0.4215, respectively.

An investigation in [23] showed that the thermal conductivity of ULCC incorporating a 0.5 volume fraction of CS was reduced by 80% compared to the conventional cementitious composite. Table 3 shows that the thermal conductivity of Composite 3 ($\lambda_{si}=0.5218$) that incorporated a 0.2236 volume fraction of CS ($VF_{Ai}+VF_{CSi}=0.1508+0.0728=0.2236)$ was reduced by 13.03% compared to CAC ($\lambda_{si}=0.61$). The thermal conductivity of Composite 2 ($\lambda_{si}=0.4215$) that incorporated a 0.3817 volume fraction of CS ($VF_{Ai}+VF_{CSi}=0.2681+0.1136=0.3817)$ was reduced by 29.75% compared to CAC ($\lambda_{si}=0.61$). The analytical approach in [23] did not consider the topology of CSs, while our numerical model proposed in this paper evaluated the position of CS.

Numerical simulation of the thermal conductivity of the MHM and composite models revealed a correlation between the thermal-conductivity value and the volume fraction of the constituent materials. Table 3 shows a slight (~4.57%) increase in the thermal conductivity of Composite 1 ($\lambda_{Si}=0.6274\;W/\mathrm{mK}$) compared to the CAC matrix ($\lambda_{Si}=0.61\;W/\mathrm{mK}$). This can be explained by the fact that the volume fraction of the CAC matrix ($VF_{CACi}$) decreased from 1.00 (in MHM2) to 0.9346 (in Composite 1), while the volume fraction of the cenosphere shell and the air was 0.0319 and 0.0335, respectively. The thermal conductivity of the cenosphere shell ($\lambda_{Si}=2.00$) was ~3.33 times larger than CAC ($\lambda_{Si}=0.61$); therefore, a more thermally conductive cenosphere shell increased the thermal conductivity of Composite 1.

The effects of CS on both the mechanical and thermal properties of LRC have been estimated [19]. CSs were studied in connection with various sizes, proportions, and wall thicknesses. It was revealed that the thermal conductivity of LRC was mainly dependent on the volume fraction (VF) of CS. The thermal conductivity of Composite 3 ($\lambda_{si}=0.5218$) that incorporated a 0.2236 volume fraction of CS decreased by 13.03%, while the TC of Composite 2 ($\lambda_{si}=0.4215$) that incorporated a 0.3817 volume fraction of CS decreased by 29.75%. Therefore, our results align with [19] since our numerical simulations confirm that thermal conductivity values decreased due to an increased volume fraction of CS in the composite samples. 

The decrease in the thermal conductivity of composites was mainly due to the thermal degradation of the cement paste phase, the change in the volume fraction of the constituent materials, the increase in micro-cracks, and the damage of the interface transition zone between the particles and the cement paste [56].

Table 3 shows that the composite samples (Composite 1, Composite 2, and Composite 3) demonstrated thermal-conductivity values of 0.6274, 0.4215, and 0.5218 W/mK at 1400 °C, respectively (Table 3). In the solid concrete matrix, the thermal conductivity value of 2.0 W/m·K was obtained numerically. The authors of [38] also investigated the thermal conductivity of ultra-lightweight cement composites with cenospheres. The results for oven-dried specimens were lowered (0.31–0.40 W/m·K) compared to the thermal conductivities of the oven-dried cement pastes (0.84 and 0.80 W/m·K). Therefore, our results align with [38] since our numerical simulations confirmed that the thermal conductivity values decreased due to an increased volume fraction of the air in the composite samples.

The first impression of the comparison of thermal conductivities was the sufficient agreement of numerical and analytical results. The highest deviation was observed for the Composite 3 model. Given that the analytical relationship (Equation (2)) appeared more complex and sophisticated, the numerical results better represented the nonlinear heat-transfer behavior. This can be explained by the assumption lying behind the analytical approach: in particular, the distribution (or topology) of air pores within the investigated domain was not considered. However, the microstructure topology of particle composites is known to play a crucial role in the heat-transfer process [58].

Composite 2 exhibited the lowest value of thermal conductivity compared to Composites 1 and 3 (Table 3) due to the significant influence of the thermal conductivity of the air inside the cenosphere. Considering Composite 3, it may be concluded that the amount of binder (i.e., matrix of CAC) in the composite should be as small as possible, and ideally, a point fusion of cenospheres could be formed with the help of a nano-volume binder. The numerical results demonstrated the contribution of porosity (air volume) and suitable technological control, which indicate new trends for numerical modeling.

## 5. Conclusions

The following conclusions summarize the presented paper:

1. Numerical, experimental, and theoretical analyses of the thermal properties of porous lightweight refractory concrete were performed. The studied sample was investigated as the combination of a calcium aluminate cement matrix, with the addition of aluminosilicate cenospheres being the main factor in a considerable reduction in thermal conductivity.
2. The simplified representation of the sample’s microstructure was obtained based on scanning electron microscopy testing. The geometrical characteristics of cenospheres—shape, dimensions, and complexity of the particle shell—were evaluated and transferred to the modeling part.
3. A finite element scheme was employed to determine the thermal properties at the microscopic scale—effective thermal conductivity and thermal stresses. Simulations were performed on geometrical models containing cenosphere particles of various sizes and numbers. The thermal stress distributions revealed that the stress concentrations occurred in the shell layer of the cenosphere.
4. The results indicated the quantitative difference in the thermal performance of the investigated models. The larger the cenosphere particle was, the greater reduction in effective thermal conductivity observed. It was found that the void/porosity level mostly governed conductivity, regardless of the shape or the distribution of particles at the microscale level.
5. Effective thermal conductivity evaluated by the numerical approach was compared with the analytical results obtained by two models derived from the theory of a two-phase mixture. It was shown that the selection of appropriate theoretical relations enabled the numerical reproduction of the correct behavior of the thermal conductivity of porous lightweight refractory concrete composites with changing porosity.
6. Numerical analysis revealed that the thermal conductivity of ALRC was mainly dependent on the volume fraction of air. The higher the volume fraction of air, the lower the thermal conductivity of ALRC achieved.
7. Despite the satisfactory agreement, more research is required concerning the real representation of sample microstructure, the dependence between the micro- and macroscopic scales, and the application of experimental testing to validate the numerical results.

## Figures and Tables

**Table 1:** The proportion of materials in the ALRC.

Material	Amount, % of Mass
Calcium aluminate cement Górkal 70	35
Aluminosilicate cenospheres	65
Water	55

**Table 2:** Homogeneous material properties at 1400 ℃ [55].

Homogeneous Material	Density, $\rho ,\;kg/m^{3}$	Heat Capacity at Constant Pressure, $C_{p},\;J/kg\cdot K$	Thermal Conductivity, λ, W/m·K
MHM1	3030.00	682.00	2.00
MHM2	2300.00	880.00	0.60
MHM3	0.20	1200.00	0.09

**Table 3:** Thermal conductivity $\lambda_{in}$, $\lambda_{si}$, and $\lambda_{eff}$, and volume fraction ($VF_{i}=V_{i}/V$ ) at 1400 ℃.

Material	Cenosphere Shell (Aluminosilicate)		Calcium Aluminate Cement (CAC)		Air		Simulated Values (Equation (3))	Effective Values (Analytical)
	$\lambda_{in}$	$VF_{CSi}$	$\lambda_{in}$	$VF_{CACi}$	$\lambda_{in}$	$VF_{Ai}$	$\;\;\;\lambda_{si}$	$\lambda_{eff}$
MHM1	2.00	1	-	-	-	-	2.00	-
MHM2	-	-	0.60	1	-	-	0.61	-
MHM3	-	-	-	-	0.09	1	0.09	-
Composite 1	2.00	0.0319	0.60	0.9346	0.09	0.0335	0.6274	0.6277 (Equation (1))
Composite 2	2.00	0.1136	0.60	0.6183	0.09	0.2681	0.4215	0.4152 (Equation (2))
Composite 3	2.00	0.0728	0.60	0.7764	0.09	0.1508	0.5218	0.5039 (Equation (3))

## Data Availability

Not applicable.
